# Patient expectation and experience of MR-guided radiotherapy using a 1.5T MR-Linac

**DOI:** 10.1016/j.tipsro.2023.100224

**Published:** 2023-11-25

**Authors:** S.R. de Mol van Otterloo, J.M. Westerhoff, T. Leer, R.H.A. Rutgers, L.T.C. Meijers, L.A. Daamen, M.P.W. Intven, H.M. Verkooijen

**Affiliations:** a Department of Radiation Oncology, University Medical Center Utrecht, Heidelberglaan 100, 3508 GA Utrecht, the Netherlands; b Division of Imaging, University Medical Center Utrecht, Utrecht, the Netherlands

**Keywords:** MR-Linac, MRgRT, Patient reported outcomes, Patient reported experience measures (PREM)

## Abstract

**Background and Purpose:**

Online adaptive MR-guided radiotherapy (MRgRT) is a relatively new form of radiotherapy treatment, delivered using a MR-Linac. It is unknown what patients expect from this treatment and whether these expectations are met. This study evaluates whether patients’ pre-treatment expectations of MRgRT are met and reports patients’ on-table experience on a 1.5 T MR-Linac.

**Materials and methods:**

All patients treated on the MR-Linac from November 2020 until April 2021, were eligible for inclusion. Patient expectation and experience were captured through questionnaires before, during, and three months after treatment. The on-table experience questionnaire included patient’ physical and psychological coping. Patient-expected side effects, participation in daily and social activity, disease outcome and, disease related symptoms were compared to post-treatment experience.

**Results:**

We included 113 patients who were primarily male (n = 100, 89 %), with a median age of 69 years (range 52–90). For on-table experience, ninety percent of patients (strongly) agreed to feeling calm during their treatment. Six and eight percent of patients found the treatment position or bed uncomfortable respectively. Twenty-eight percent of patients felt tingling sensations during treatment. After treatment, 79 % of patients’ expectations were met. Most patients experienced an (better than) expected level of side effects (75 %), participation in daily- (83 %) and social activity (86 %) and symptoms (78 %). However, 33 % expected more treatment efficacy than experienced.

**Conclusion:**

Treatment on the 1.5 T MR-Linac is well tolerated and meets patient expectations. Despite the fact that some patients expected greater treatment efficacy and the frequent occurrence of tingling sensations during treatment, most patient experiences were comparable or better than previously expected.

## Highlights

• One of the first studies reporting on patient expectation and their experiences of online adaptive MR-guided radiotherapy (MRgRT) using a high field MR-Linac.
• Treatment was generally well tolerated and on-table experience was good.
• Patient expectation was high and most of them were met by the post treatment experience.

## Introduction

Radiotherapy is used as a curative or palliative treatment in approximately half of the cancer patients [1], [2], [3]. Unfortunately, radiotherapy is also associated with toxicity. This is caused by radiation exposure of healthy tissues and organs at risk (OAR) surrounding the tumor [4], [5]. Traditionally, large treatment fields were used to compensate position uncertainties of the tumor and surrounding structures during and between delivery of each fraction [6], [7], [8], [9].

The 1.5 Tesla (T) Magnetic Resonance Linear accelerator (MR-Linac), a combination of a linear accelerator and a 1.5 T MR-scanner, was introduced into clinical practice to reduce the position unceartainties [10], [11]. This device enables MRI-guided radiotherapy (MRgRT) according to an online adaptive workflow based on MR images [12]. The online workflow enables real time visualization at each fraction and thereby allows a decrease of the radiation field size by reduction of position uncertainties during each treatment fraction [13], [14]. The MR-Linac holds the promise to reduce toxicity and enables dose-escalation strategies in multiple tumor sites [15], [16], [17], [18], [19].

The online adaptive workflow on the MR-Linac uses multiple MR acquisitions and delineation of patient’ anatomy at each fraction, which increases the treatment duration as compared to treatment with conventional, non-adaptive devices. This requires patients to lie still and hold treatment positions longer, which might adversely impact patient experience [14], [20]. Also, informing patients about the assumed benefits of MR-Linac (e.g. smaller margins thus reduced toxicity) might raise patient expectation regarding their treatment outcomes. If these pretreatment expectations are not met, patients may be disappointed and this may negatively affect their experience [21], [22].

This study aims to explore the on-table patient reported experience of patients undergoing high field MR-Linac treatment and to compare patient expectation of MR-Linac treatment outcomes (eg. including toxicity, disease-related symptoms, treatment results and participation in social and daily activities) to their actual experience.

## Materials and methods

All patients treated on a 1.5 T MR-Linac (Unity, Elekta AB, Stockholm, Sweden) at the University Medical Centre (UMC) Utrecht between November 2020 and April 2021, who consented to the ‘Multi-OutcoMe EvaluatioN of radiation Therapy Using the MR-Linac’ (MOMENTUM) study were eligible. Inclusion was independent of tumor site or intention of treatment as long as the scheduled treatment consisted of three or more fractions. Moreover, the MR-Linac is equipped with two adaptation protocols: an adapt-to-shape (ATS) and an adapt-to-position (ATP) protocol [12]. Patients were included regardless of the adaptation strategy used during their treatment. Further, patients were included regardless of receiving concomitant (chemo)therapy as the aim of this prospective cohort was to reflect everyday practice. The MOMENTUM study was approved by the Medical Research Ethics Committee of the UMC Utrecht in the Netherlands [23]. Patients consented to participate in the current study separately.

Patients were surveyed at three points in time: before the first fraction (Q1), during treatment (directly after the third or fourth fraction on the MR-Linac) (Q2), and at three months after completion of treatment (Q3). After consent, patients could respond to Q1 at any time prior to the first fraction. Q2 was filled out at our institution directly after the treatment. Patients received information on both conventional and MR-Linac treatment after which the treatment modality was chosen based on shared decision-making.

At Q1, patient’ expectation was assessed by a questionnaire developed and piloted at the UMC Utrecht, including questions on patient expectation regarding side effects, disease-related symptoms, impact of MR-Linac treatment on participation in social- and daily activities, and disease course. Expected disease course was to be interpreted by the patient with examples of improved tumor (marker) response to treatment, for instance tumor shrinkage, downstaging and/or decreased tumor marker (e.g. Prostate Specific Antigen (PSA) for prostate tumors). At Q3, patients were asked to complete a similar questionnaire, in which they could indicate their actual experience regarding the same items. This questionnaire was sent to their home or email address.

For the on-table experience (Q2), we adapted the questionnaire capturing patient experience of MRgRT on the 1.5 T MR-Linac developed by Barnes et al. by adding a ‘neutral’ option to the responses scoring items on a 5-point Likert scale [24]. Scores were post-processed for negatively phrased questions so that high scores (1,2) were attributed to a positive response and low scores (-2,-1) to a less positive response across all questions. For in-depth analysis of the most frequently reported negative item, patients were contacted by phone.

Patients were given the option to complete questionnaires either on-paper or digitally. Two months after start of the study, a nationwide lockdown due to the SARS-CoV-2 (COVID-19) pandemic required us to fully digitalize the questionnaires. Thereafter patients could receive digital questionnaires only.

Patient characteristics and treatment details were extracted from the MOMENTUM registry [23.] Data was presented using frequencies and proportions for categorical data and means with standard deviation or medians with interquartile ranges (IQR) for continuous data. Descriptive statistics were used to evaluate patient pre-treatment expectation, on-table experience, and post-treatment experience by using proportions and means with standard deviations. For comparison of pre-treatment expectations vs. post-treatment experiences we used complete cases, i.e. patients that filled in the Q1 *and* Q3 surveys. For sensitivity analysis we reviewed all available questionnaires (Supplements). Statistical Package for Social Sciences (SPSS) software version 25 was used for analysis.

## Results

**Fig. 1:**
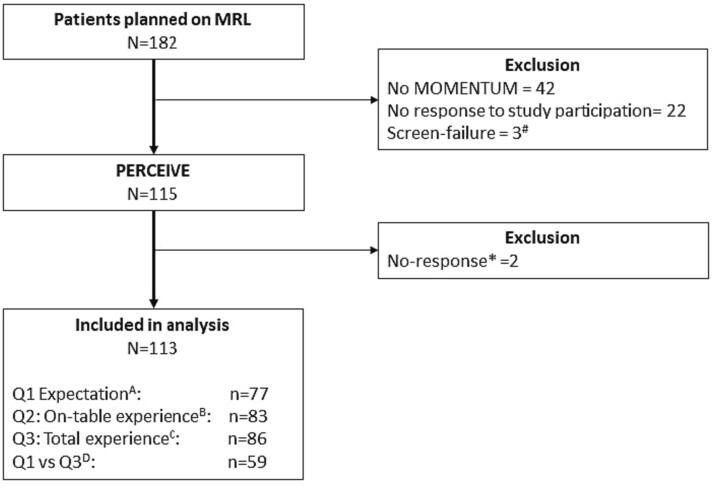
**Flowchart of patients included for analysis after in- and exclusion criteria.***Abbreviations: MOMENTUM = the The Multi-OutcoMe EvaluatioN of radiation Therapy Using the Unity MR-Linac Study, MRL = MR-Linac, QoL = Quality of Life.A Q1 Expectation: Questionnaire capturing pre-treatment expectation of patients.B Q2 On-Table Experience: Questionnaire capturing on-table experience of patients after 3rd fraction.C Q3 Total experience: Questionnaire capturing the totals experience of the treatment 3 months after treatment.D Q1* vs *Q3: patients that completed Q1 and Q3 questionnaires.^#^ Patients who had their first fraction and before receiving the first questionnaire were considered screen failure.*

In total, 182 patients were treated on the MR-Linac during the inclusion period (Fig. 1). Of these, 42 patients did not consent for participation in the MOMENTUM study and 3 had not received the first questionnaire before their first treatment (screen-failure). Moreover, twenty-two patients were contacted for study participation but did not respond. Of 115 patients who consented to PERCEIVE, two patients did not respond to the questionnaires. Therefore, 113 patients were included in our study. Seventy-seven patients (68 %) responded to Q1, 83 patients (73 %) to Q2 and 86 patients (76 %) responded to Q3. A total of 59 patients (52 %) responded to both Q1 and Q3.

The study population (n = 113) consisted mainly of male patients (89 %) who were treated for prostate cancer (64 %) (Table 1). Other common treatment indications were oligo lymph node metastases (14 %) and pancreatic cancer (7 %). Most patients were treated with curative intent (75 %) and through an ATS protocol (84 %). Nine patients (8 %) received chemo- or immunotherapy during or within three months after MR-Linac treatment.

**Table 1:** Patient characteristics, clinical indications and details of MR-Linac treatment.

		N (%)
**Total patients**		113
**Gender**	Male	100 (89 %)
**Age** (median (range))		69 (52–90)
**Patients per tumor site**	Prostate	72 (64 %)
	Oligo lymph node	16 (14 %)
	Pancreas	8 (7 %)
	Lung	8 (7 %)
	Rectum	4 (4 %)
	Esophagus	5 (4 %)
	Other*	6 (5 %)
**Concurrent treatment^$^** (n = 21)	Chemotherapy	8 (17 %)
	Immunotherapy	1 (1 %)
	Hormonetherapy	11 (10 %)
**Delineation protocol**	Adapt-To-Shape Adapt-To-Position Mixed Unknown	95 (84 %)6 (5 %)1 (1 %)11 (10 %)
**Treatment intent**	Curative	85 (75 %)
	Palliative	28 (25 %)
**Delivered fractions on MR-linac** (median (range)		5 (3–20)
**Good performance score^** (n = 86)		74 (86 %)

* Other includes Uterus, vaginal, hepaticobilliairy, Lanyngeal and urethral cancer patients.

*$ Other treatments concurrent with MR-Linac treatment or that started within the 3 months thereafter for the 21 patients with known data.^ Good performance score defined as: KPS ≥ 80 %, ECOG 0, CCI ≤ 3.*

## Overall pre-treatment expectations (Q1)

Of the 77 patients that responded to Q1, 41 patients were hopeful or expectant prior to MR-Linac treatment. Thirty patients felt privileged or special, 23 patients were neutral about being treated on the MR-Linac, and one patient was content. Nine patients felt nervous or tensed, four patients were uncertain or worried and two patients felt anxious before treatment.

## On-table experience (Q2)

**Fig. 2:**
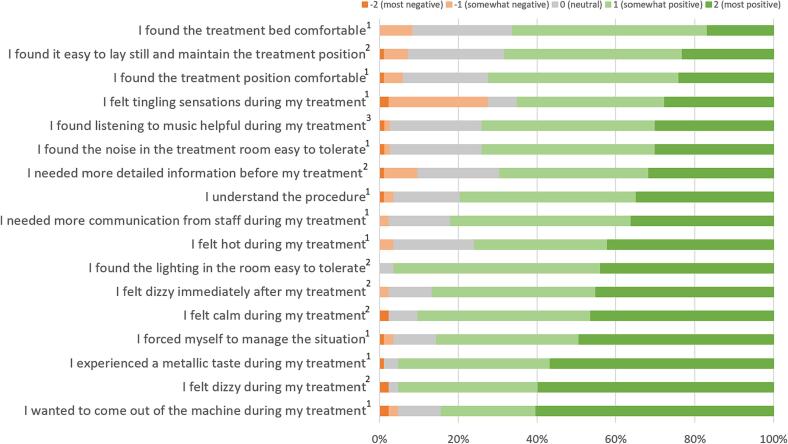
**Patient on-table experience of their MR-Linac treatment stratified into positive and negative answers.***1 = 82 patients answered the questions (n = 82)0.2 = 83 patients answered the questions (n = 83)0.3 = 73 patients listened to music and answered the question (n = 73) Questions are ranked from smallest to most amount of ‘most positive’ patients.*

Of the 83 patients that completed Q2, the majority was calm during treatment and managed the situation well (Fig. 2). Treatment position and bed were reported to be uncomfortable by five and seven patients, respectively. Six patients had difficulties with maintaining the treatment position.

Twenty-three patients felt tingling sensations during treatment. Fifteen patients who reported this were contacted by phone after 12 months. Eight patients could not remember having these sensations. The remaining seven patients reported to have experienced localized tingling in their limbs during treatment. None of these patients experienced these sensations as painful and two patients described them as a nuisance. Five patients experienced the tingling sensations during several fractions and two patients felt them during all fractions.

Twelve patients provided additional comments (open text field) at Q2, mostly commenting on issues captured in the questionnaire as mentioned above. In addition, one patient had difficulties with communication during the procedure due to a low sound volume, one patient noted a cold breeze, and two patients reported the need to get used to the treatment. Two patients were satisfied: one reported not noticing the treatment and another complimented the team.

## Overall post-treatment experience

Of 86 patients that completed Q3, 68 patients reported that their pre-treatment expectations were met. Fourteen patients were uncertain about whether their expectations were met, as they had not yet visited their physician or did not know their post-treatment PSA level, and four patients reported that their expectations were not met. Of those four patients, two patients reported a worse experience than expected, as their serum PSA levels had not decreased. One patient experienced more side effects than anticipated. For the remaining patient, the reason for unmet expectations was unknown. Of the 86 patients, seventy-eight patients would choose the MR-Linac for future radiotherapy if needed. The other eight patients were indifferent of whether they would choose the MR-Linac again. None of the patients rejected the MR-Linac for future treatment.

## Expectations vs experience of treatment outcomes (Q1 vs Q3)

**Fig. 3:**
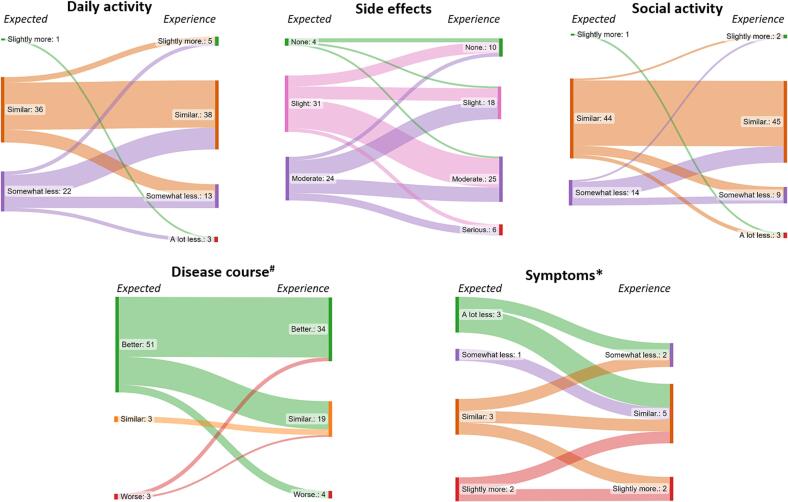
**Flow of patient expectation and experience of their high field MR-Linac treatment.***Flow for 59 patients from expectation before MR-Linac treatment on the right to experience after MR-Linac treatment on the left. Fifty-seven, nine, and fifty eight patients responded to the questions on disease course, disease related symptoms and social activities respectively.*

**Fig. 4:**
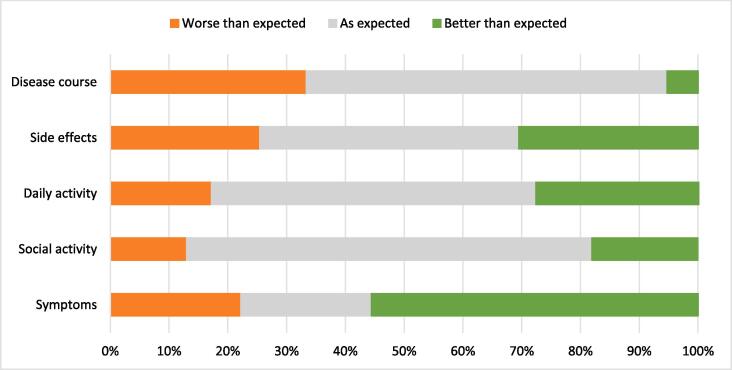
**Expectation versus experience for 59 patients treated on an MR-Linac.***Fifty-seven, nine, and fifty eight patients responded to the questions on disease course, disease related symptoms and social activities, respectively.*

Patients’ expectations before, and actual experience after treatment are shown in Fig. 3. Of 59 patients who completed both Q1 and Q3 questionnaires, it was shown that 15 patients experienced worse side effects than expected (Fig. 4). In 26 patients post-treatment experience with regard to side effects was as expected, and eighteen patients experienced less side effects than expected (Fig. 4).

Fifty-seven patients responded to the question on disease course. Of those, nineteen patients experienced a worse disease course than expected. Post-treatment disease course was as expected in 35 patients, and for three patient the disease course was better than expected.

Ten patients experienced poorer participation in daily activities than expected. For seventeen patients, actual participation was better than they expected.

After treatment, eight patients (participated less in social activities than anticipated whereas pre-treatment expectations with regard to social activities were met in 40 patients. For eleven patients, participation in social activities exceeded their expected participation.

In nine patients who experienced disease-related symptoms prior to and after treatment, five patients experienced less symptoms than anticipated. In two patients, severity of symptoms was as expected, and two patients experienced worse symptoms than expected.

Finally, 34 patients reported a better experience than expected, whilst 33 patients reported a worse experience than expected on one or more questions. When comparing baseline characteristics of those groups (Table 2), patients with a better response contained more men (94 % vs 88 %).

**Table 2:** Patient characteristics of patients with a better or worse experience than expected.

		N (%)
Patients with better experience*		N = 34
**Age** (median (range))		73 (54–86)
**Gender**	Male	32 (94 %)
**Patients per tumor site**	Prostate	24 (70.6 %)
	Oligo lymph node	5 (14.7 %)
	Pancreas	3 (8.8 %)
	Oropharynx	1 (2.9 %)
	Ureter	1 (2.9 %)
Patients with worse experience*		N = 33
**Age** (median (range))		71 (54–81)
**Gender**	Male	29 (88 %)
**Patients per tumor site**	Prostate	22 (67.6 %)
	Oligo lymph node	4 (12.1 %)
	Pancreas	2 (6.1 %)
	Rectum	2 (6.1 %)
	Esophagus	1 (3.0 %)
	Lung	1 (3.0 %)
	Ureter	1 (3.0 %)

*Patients reported a better or worse experience on one or more of the questions regarding the impact of MR-Linac treatment on side effects, disease-related symptoms, participation in social- and daily activities, and the disease course.

## Review of all questionnaires (Q1 and Q3)

Sensitivity analysis comparing the 77 pre-treatment and 86 post-treatment questionnaires, showed that overall expectation and experience resembled previous results (Supplements).

## Discussion

This study showed that most patients have high expectations of MR-Linac treatment with regards to toxicity, disease-related symptoms, treatment results, and participation in social and daily activities. Expectations were generally met or exceeded by their post-treatment experience. In addition, the overall on-table patient experience was good, as > 90 % of patients reported to be (more than) content with the treatment table, treatment position and in-room facilities. Noticeable is that a relatively large number of patients experienced tingling sensations during treatment. However, considering that most patients did not found them a nuisance or could not recall the sensations during follow-up, clinical relevance of this finding is debatable.

During the first years after implementation of the MR-Linac, the majority of studies focused on treatment- and disease-related outcomes. Moreover, previous studies evaluated on-table experience on low field MR-Linacs (MRidian, Viewray Inc, Oakwood, USA), included small number of patients, or defined patient comfort as ‘not quitting treatment’ [23], [25], [26], [27]. Patient experience, however, comprises more than on-table experience, including experience of side effects or change in disease-related symptoms after treatment, as well as the extent to which the patient expectation is met. Three studies previously reported on patterns of care, using the high or low field MR-Linac, and concluded that patients tolerated MRgRT considering that no patient stopped treatment due to discomfort [27], [25], [28]. In-depth analysis of patient experience is, however, missing. Two large studies (n = 90 and n = 150) reported on patient experience by focusing solely on the on-table experience during MRgRT, and four other studies reported on on-table experience as a secondary aim [29], [30]. 1 In line with our results, all studies reported overall good patient experience. However, compared to our study, patients scored less positively on room temperature, noise, and treatment duration in all of these studies [31], [32], [33], [34]. This might be explained by the precautionary measures taken at our facility e.g. covering patients with blankets to prevent them from feeling cold during their treatment. Furthermore, Klüter et al. and Sayan et al. compared the on-table experience during the first fraction to the on-table experience during fractions later in the treatment course [30], [26]. These studies showed no significant difference in patient experience with regards to treatment table, treatment position or in-room facilities. Only anxiety during treatment was significantly reduced during later fractions [30]. In our study only two patients (2 %) totally disagreed with feeling calm during treatment and four patients (5 %) felt that they wanted to get out of the MR-Linac. As we measured the on-table experience only once, after the third or fourth fraction, we do not know whether these number decreased over time as seen by Klüter and Sayan, which might warrant further investigation. However, it is reassuring that these anxiety-related outcomes were only reported by a small number of patients. To the best of our knowledge, this is the first study to report on patient expectations prior to treatment on a high field (1.5 T) MR-Linac (Elekta, Stockholm Sweden) and the actual patient experience beyond on-table treatment experience. Interestingly, our study found that some patients experienced tingling sensations. One study investigating patient experience on a low-field MRgRT device (MRIdian, ViewRay, Inc., Mountain View, CA) found similar results, with 28 % of patients (n = 42) reporting tingling sensations. In their study, this symptom was correlated to the use of breath-holds (p = 0.027) [29]. Sayan et al. also reported tingling sensations in 57 % of patients (n = 90) using a low-field MR-Linac and in the study of Barnes et al., 40 % of patients reported moderate tingling sensations during treatment [24]. Several theories might explain the occurrence of tingling sensations [30]. Tingling can arise from unnatural treatment positions, e.g. when patients have to hold their hands over their head or chest. Also, tingling sensations might occur due to the lengthy duration of MRgRT treatment sessions. Theoretically, staying in unnatural positions during treatment can cause slight vessel entrapment inducing said tingling sensations. Moreover it is known that the magnetic field gradients from the MRI can cause peripheral nerve stimulation [35]. For our patients these tingling sensations were transient and most patients did not find them a nuisance. However to prevent or alleviate tingling, the underlying cause must be investigated. Future patient-experience studies should be focused on these sensations to improve patient experience even further.

This study shows that patient expectation is generally high for MR-Linac treatment. It indeed has been described that cancer patients have high hopes and expectations of their (chemotherapy) treatment and are generally optimistic about their prognosis [36], [37]. It is understandable that innovations in cancer treatment, that aim to improve oncological outcomes, render hope in treated patients. It is important that physicians are aware of this. On the other hand, it has been suggested that a positive mindset improves health related Quality of Life (QoL) in advanced cancer patients [38]. A positive attitude towards post-treatment outcomes could therefore potentially improve patients’ QoL. Ultimately, treating physicians have the challenging task to set optimistic yet realistic expectations before treatment so to gain possible QoL yet prevent disappointment after treatment.

In our study, most patients’ expectation were met or exceeded by the actual post-treatment experience. Treatment experience was worse than expected for 13 % − 33 % based on the domain. This might be explained by the high expectations that were reported. Inquiring patients on their expectations and addressing those when set too high might be one way to improve patient experience. However, although reported experience was lower for up to one third of patients for some outcomes, almost all patients reported that they would choose the MR-Linac for future treatments, if ever needed. These equivocal results could indicate that the pre-defined difference in expectation and experience might not be as clinically relevant as we initially thought.

Patient responses regarding on-table experience showed that they are pleased with the in-room facilities. They reported to be positive or neutral towards the comfort of the treatment bed, the treatment position, surrounding noise and in-room lighting. Before implementation of the MR-Linac, physicians were concerned that patients would have difficulties with lying still in the treatment machine and holding treatment positions for a relative long time. Nevertheless, our results indicate otherwise. These positive findings might be explained by the fact that patients are informed about potential discomfort resulting from a hard bed, uncomfortable position, and longer treatment times prior to MR-Linac treatment. This allows patients to anticipate on these inconveniences and lower their expectations, which might result in a positive experience. Another possibility is that the treatment bed is not as uncomfortable as we had anticipated. As it is expected that treatment times will only decrease with increased experience from the staff and technical advancements in MRgRT, patient on-table experience might further improve.

Our study has several limitations. Firstly the study population consists primarily of male patients, as most included patients were treated for prostate cancer. For prostate cancer, treatment duration for each fraction is relatively short, which might positively impact our study results. However, all eligible patients were asked to participate in our study, irrespective of tumor site or treatment intent. This cohort, as well as our findings, is therefore a reflection of everyday practice and applicable to routine care. Secondly, we have not stratified for the different tumors. Treatment times and protocols are different per tumor type which can impact patient experience. Thirdly, patients were included regardless of the adaptation protocol used during their treatment. However, when full-treatment adaptation is used on a daily basis, during the ATS workflow, treatment times are generally longer, than when a ATP strategy is used. It is likely that these longer treatment times affect treatment patient experience. As this study’s main objective was to report on the patient experience of MR-Linac patients treated according to (inter)national protocols, it does not include time analysis of the treatment and more research is needed to evaluate whether there actually is a difference in patient experience between the ATS and ATP protocol. Finally, the start of this study coincided with the first COVID-19 outbreak. This required adaptation of the study protocol such as a prompt switch from an in-person patient accrual to an off-site process which caused a stop in the patient accrual and thereby a reduction of our total study population. The pandemic also influenced the lives of patients as the Dutch population had to face several lockdowns. During these lockdowns participation of social and daily activities were reduced to a minimum or even forbidden through curfews and a maximum number of contacts per day. These restrictions most likely reduced the reported social and daily activities in this study. The pandemic also forced us to switch from digital and paper questionnaires to an online-only strategy.

In conclusion, MR-Linac treatment in general meets the patients’ pre-treatment expectations with regard to toxicity, disease-related symptoms, treatment results, and participation in social and daily activities. Some patients, however, expected a larger improvement of their disease course. In addition, the overall on-table patient experience during MR-Linac treatment is good, although patients can experience tingling sensations during treatment. This needs to be examined in future research to improve patient experience even further.

## Declaration of Competing Interest

The authors declare that they have no known competing financial interests or personal relationships that could have appeared to influence the work reported in this paper.
