# Supplementary material for: Patient expectation and experience of MR-guided radiotherapy using a 1.5T MR-Linac
Source: Tech Innov Patient Support Radiat Oncol. 2023 Nov 25;29:100224. doi: 10.1016/j.tipsro.2023.100224 (PMC10755768; doi:10.1016/j.tipsro.2023.100224)

**Supplements**

Figure depicting expectation (n=77) and experience (n=86) for all patients in analysis in percentages.


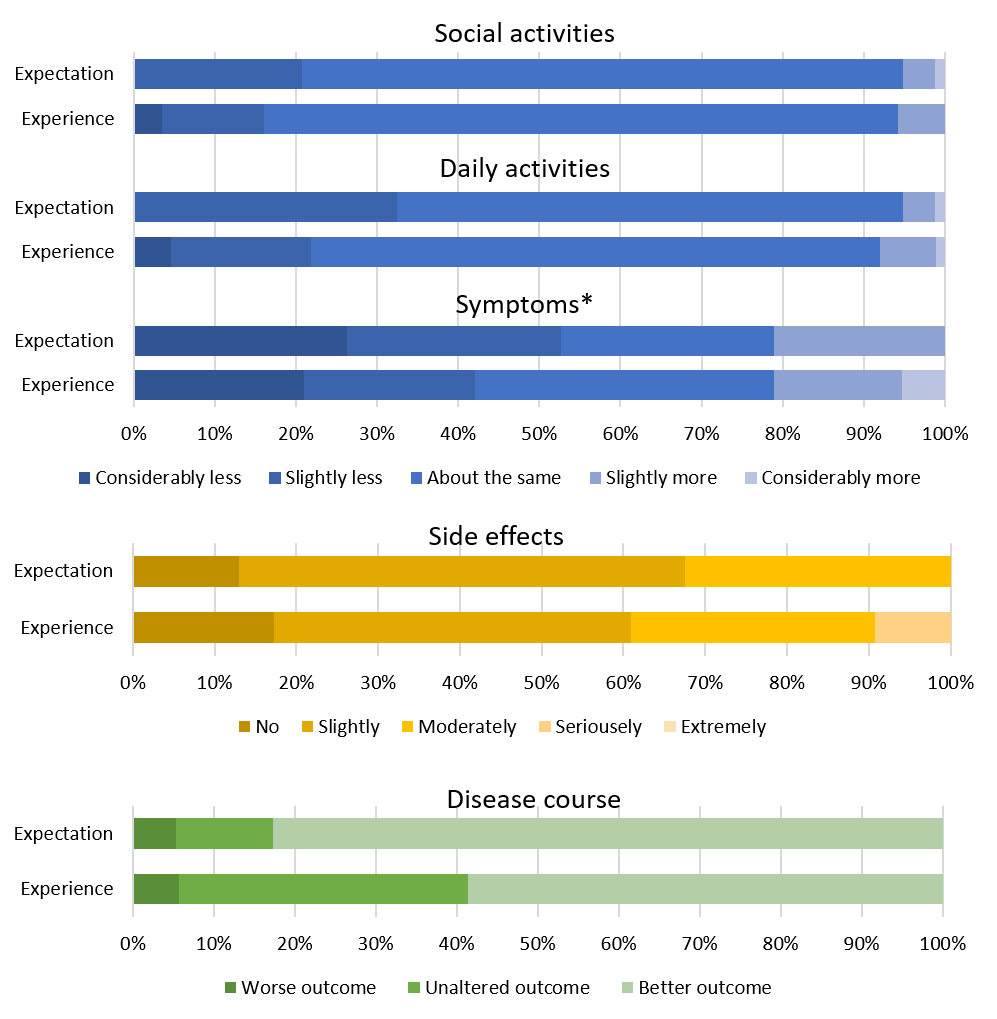

Supplement: Supplementary data 1 [file mmc1.docx]
